# Biglycan enhances gastric cancer invasion by activating FAK signaling pathway

**DOI:** 10.18632/oncotarget.1871

**Published:** 2014-03-26

**Authors:** Lei Hu, Yan-tao Duan, Jian-fang Li, Li-ping Su, Min Yan, Zheng-gang Zhu, Bing-ya Liu, Qiu-meng Yang

**Affiliations:** ^1^ Shanghai Key Laboratory of Gastric Neoplasms, Shanghai Institute of Digestive Surgery, Ruijin Hospital, Shanghai Jiao Tong University School of Medicine, Shanghai, People's Republic of China.

**Keywords:** BGN, Gastric cancer, Metastasis, FAK

## Abstract

Biglycan (BGN) is an important member of small leucine-rich proteoglycans family, and has been implicated in oncogenesis and development of various human cancer types. Here we report that BGN promotes tumor invasion and metastasis of gastric cancer both *in vitro* and *in vivo*. BGN expression is significantly higher in gastric cancer tissues and associated with lymph node metastasis, depth of tumor invasion and TNM stage. BGN enhances gastric cancer cell wound healing, migration and invasion ability as well as the tube formation ability of endothelial cells *in vitro*. Animal experiments results *in vivo* are consistent with outcomes *in vitro*. BGN induces increased phosphorylation of FAK (Tyr576/577, Tyr925 and Tyr397) and Paxillin. These results indicate that BGN is upregulated, and plays an oncogenic role, in gastric cancer metastasis by activating the FAK signaling pathway.

## INTRODUCTION

Gastric cancer is one of the most common malignant tumors worldwide, especially in East Asia ( China, Japan, and Korea) [1]. Early-stage gastric cancer is usually asymptomatic or associated with nonspecific symptoms, such as dyspepsia. By the time symptoms occur, it has often reached an advanced stage. The 5-year survival rate is only 20–40% after surgery [2]. Thus, discovery of new biomarkers and wider insight into the mechanisms involved in gastric tumorigenesis and metastasis are crucial. Tumor metastasis is a series of discrete sequential events in a biological cascade: First, carcinoma cells usually show decreased intercellular adhesiveness and increased motility and invasiveness. They can then disrupt the basement membrane and extracellular matrix (ECM) to enter and survive in the circulation. Finally, cancer cells arrive at and colonize distant tissue [3, 4].

ECM is a critical modulator of epithelial cell morphology and migration, and of differentiation of a wide array of tissue types [5]. BGN is an ECM protein that belongs to the family of small leucine-rich proteoglycans [6]. BGN has been found in almost every organ within human body, but it is not uniformly distributed within each organ. BGN was found to be expressed on the cell surface and sometimes within the extracellular matrices of a range of specialized cell types [7]. Recent studies have indicated significantly higher expression of BGN in tumor tissues compared with adjacent normal tissues, in several cancers, including endometrial cancer [8], pancreatic cancer [9], colon tumor [10] and tumor blood vessels [11] and esophageal squamous cell carcinoma [12]. Abnormal BGN expression in tumor tissues suggests that BGN is significant in cancer pathogenesis and progression. In this study, we first analyzed expression of BGN in gastric cancer tissues using qRT-PCR and immunohistochemistry. Subsequently, we examined the effects of BGN on tumor invasion and metastasis *in vitro* and *vivo*.

## RESULTS

### BGN expression is upregulated in gastric cancer tissues

**Fig.1:**
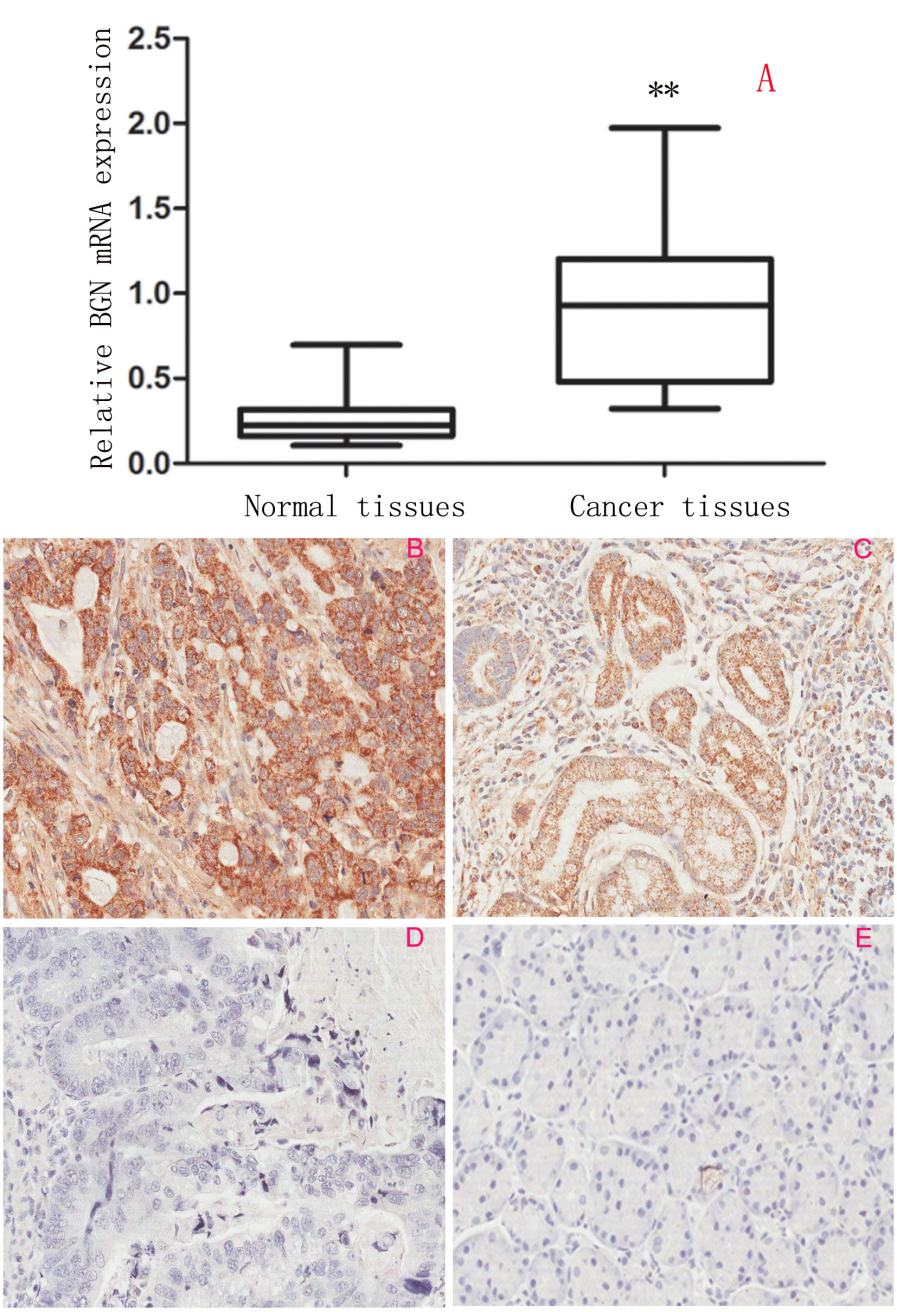
Expression of BGN in clinical gastric tissues (200×) (A) *BGN* mRNA expression in gastric cancer tissues and paired adjacent non-tumor tissues examined by qRT-PCR and normalized to *GAPDH*.(B-E) Characterization of BGN protein expression in human gastric cancer tissues and paired adjacent non-tumor tissues by immunohistochemistry staining. (B) Strong positive BGN expression in gastric cancer. (C) Weak positive BGN expression in gastric cancer. (D) Negative BGN expression in gastric cancer. (E) Negative BGN expression in non-tumor gastric mucosa

We first examined the expression of *BGN* using qRT-PCR in gastric cancer and adjacent non-tumor tissues. Gastric cancer tissues showed marked higher levels than did corresponding non-tumor tissues (*P*<0.0001, Fig.1A). Immunohistochemistry staining (Fig.1B, C, D, E) showed BGN expression was higher in tumor tissues than that in non-tumors tissues, and showed that BGN is mainly located in cytoplasm of epithelial cells. This result was consistent with mRNA expression in tumor tissues and non-tumor tissues. BGN was up-regulated in 77.8% (95/122) of gastric cancer patients. We next investigated the relationship between BGN expression and clinicopathologic features of gastric cancer, and found that up-regulated BGN was associated with lymph node metastasis (*P*=0.004), depth of invasion (*P*=0.034) and TNM stage (*P*=0.022), but not with other clinicopathological factors including sex, age, tumor location etc. (Table 1).

**Table 1:** Association between BGN expression and clinicopathological factors of gastric cancer patients

Variables	Number of cases	BGN immunostaining		*P*
		Positive(n=95)	Negative(n=27)	
Gender				
Male	92	73	19	0.491
Female	30	22	8	
Age(years)				
≥65	29	22	7	0.766
<65	93	73	20	
Tumor differentiation				
Well to moderate	43	36	7	0.251
Poor	79	59	20	
Tumor location				
Gastric fundus	5	3	2	0.226
Gastric corpus	57	48	9	
Pylorus	60	44	16	
Tumor size				
≤5m	76	57	19	0.327
>5m	46	38	8	
T stage				
T1+T2	27	17	10	0.034
T3+T4	95	78	17	
Lymph node metastasis				
Negative	40	25	15	0.004
Positive	82	70	12	
Distant metastasis				
Negative	117	90	27	0.223
Positive	5	5	0	
TNM stage				
I+II	49	33	16	0.022
III+IV	73	62	11	

Note: Positive BGN expression included all positive cases, such as weak and strong.

### BGN accelerates wound healing of gastric cancer cells

BGN expression was significantly increased in SGC-7901/BGN and MKN-45/BGN cells, and it was efficiently knocked down in SGC-7901/BGN/sh1, MKN-45/BGN/sh1 and NCL-N87/sh1-BGN, NCL-N87/sh2-BGN cells (Fig.S1A). Immunofluorescence analysis showed that BGN expression in SGC-7901/BGN and MKN-45/BGN cells was mainly distributed in cytoplasm, with no obvious positive signal in cell nuclei (Fig.S1B).

**Fig.2:**
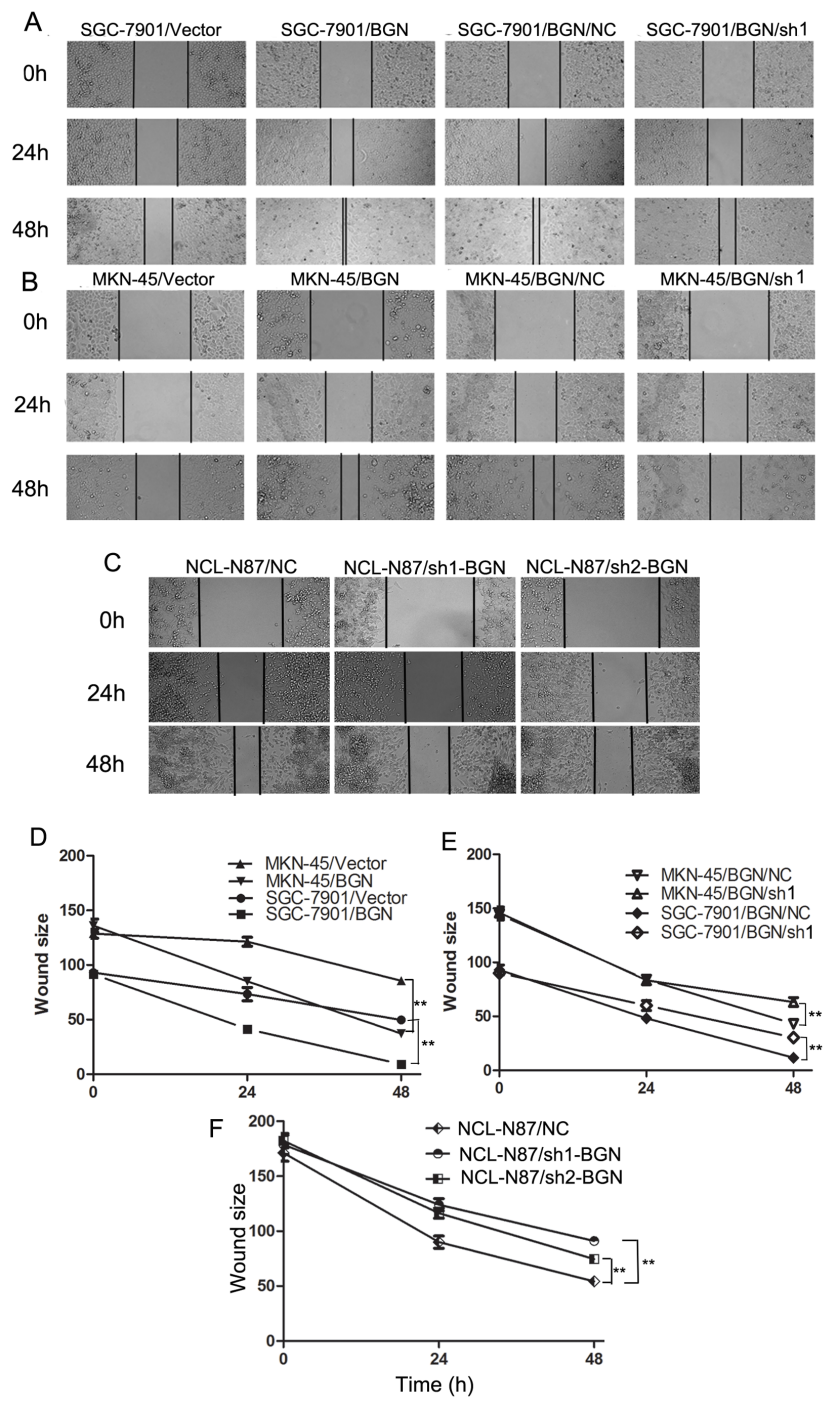
Effects of BGN on wound healing in gastric cancer cells (200×) (A, B, C) Wound healing assay with gastric cancer cells. Microscopic observations were recorded 0, 24 and 48 hours after scratching the cell surface. A representative image from every independent experiment is shown. (D, E, F) The distances between wound edges of gastric cancer cells at 0, 24 and 48 hours. *P<0.05; **P<0.01.

To investigate the effect of BGN on movement ability and wound healing, we performed a wound healing assay, in which we observed that the distance between wound edges of SGC-7901/BGN and MKN-45/BGN cells was markedly shorter than those of SGC-7901/Vector or MKN-45/Vector cells (Fig.2A, B, D, E). Additionally, we observed longer distance in wound healing of SGC-7901/BGN/sh1, MKN-45/BGN/sh1, NCL-N87/sh1-BGN, and NCL-N87/sh2-BGN cells compared with control groups (Fig.2A–F).

### BGN promotes the migration and invasion of gastric cancer cells

**Fig.3:**
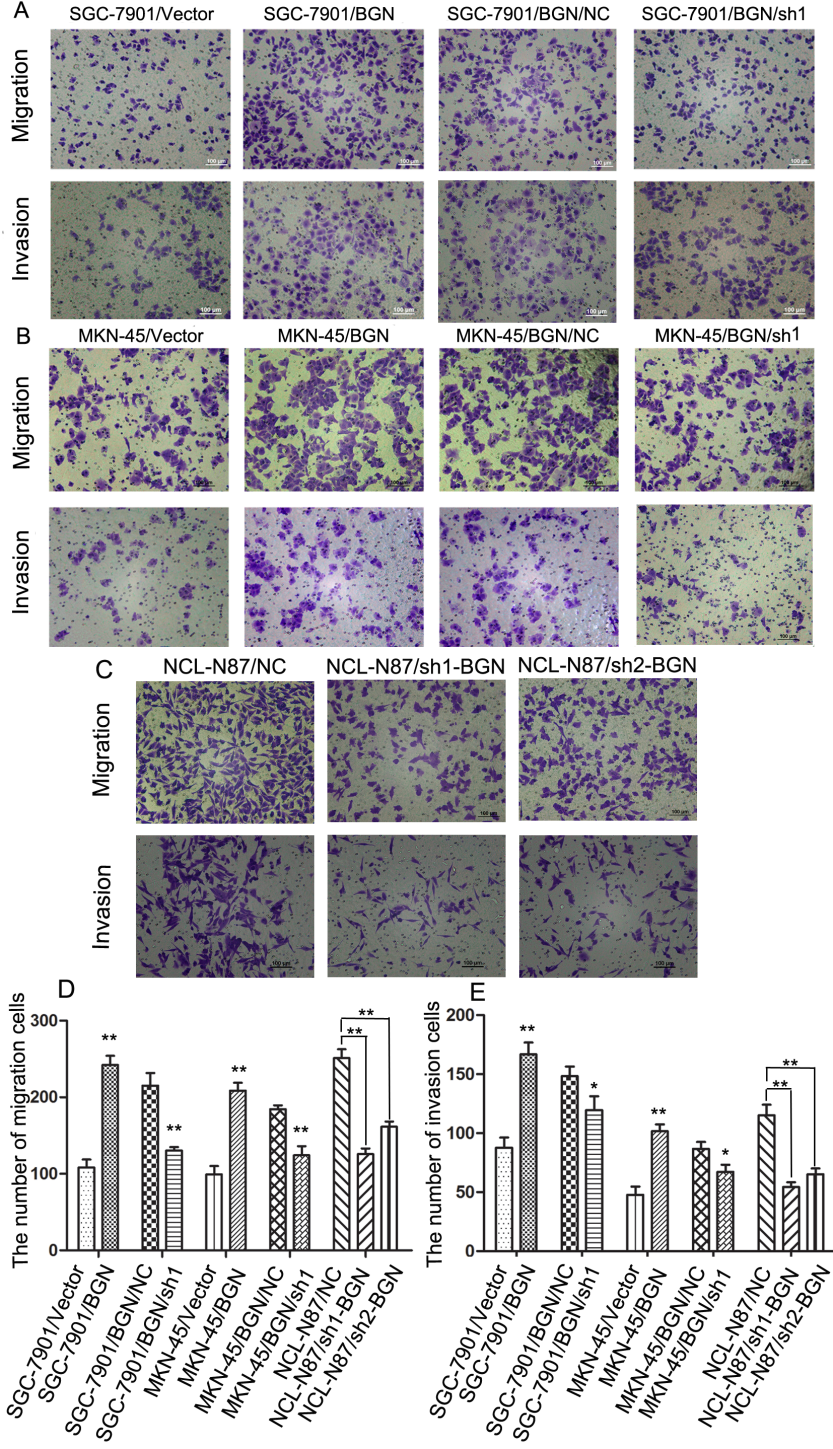
Effects of BGN on migration, invasion ability in gastric cancer cells (200×) (A, B, C) Transwell assay. Photographs show cells that travelled through the micropore membrane with or without Matrigel. (D, E) Histograms showed the numbers of migration cells and invasion cells. *P<0.05; **P<0.01.

We observed that the cell migration rate and invasion rate were both increased in BGN overexpressing cells compared with their respective control cells (Fig. 3A, B, D, E). The opposite results were obtained after BGN was knocked down in the stable cells (Fig.3A, B, D, E). Endogenous BGN was knocked down in NCL-N87 cells and showed consistent results with SGC-7901/BGN/sh1 and MKN-45/BGN/sh1 cells (Fig.3C, D, E). The experimental groups and their control groups differed significantly (*P*<0.05). In accordance with these observations, we found that phosphorylation of metastasis-related proteins, such as FAK and paxillin, were upregulated in SGC-7901/BGN cells (Fig. 5). The reverse protein level change was observed after *BGN* was knocked down in NCL-N87 cells (Fig.S2). These results suggest a functional role for BGN in gastric cancer metastasis.

### Overexpression of BGN in gastric cancer cells stimulate tubular formation in HUVECs

**Fig.4:**
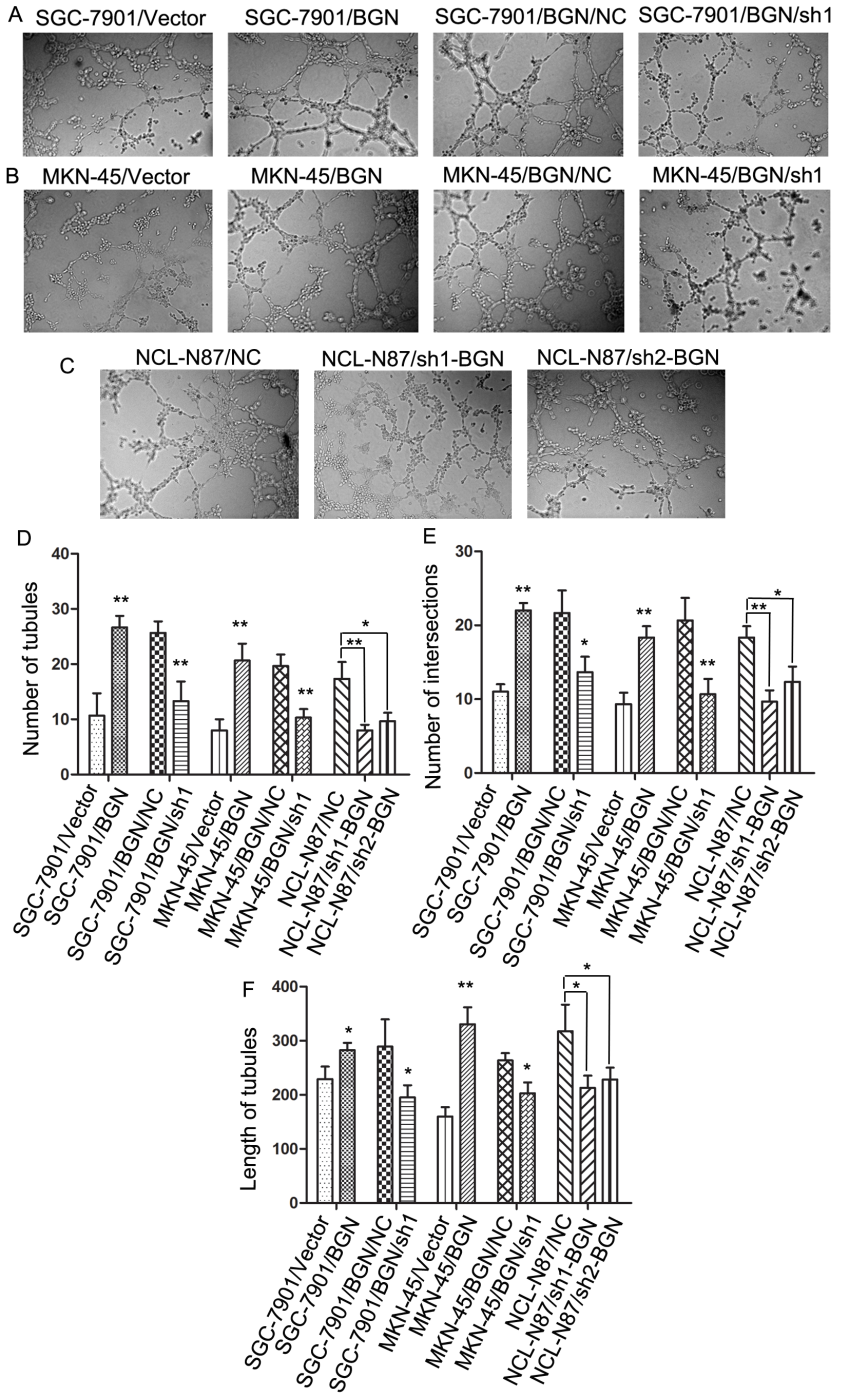
Effect of BGN on tubular formation *in vitro* (200×) (A, B, C) Richly formed tubular structure was observed in the SCG-7901/BGN and MKN-45/BGN groups compared with the SGC-7901/Vector and MKN-45/Vector groups; whereas the SCG-7901/BGN/sh1, MKN-45/BGN/sh1, NCL-N87/sh1-BGN and NCL-N87/sh2-BGN groups formed less tubular structures than the SCG-7901/BGN/NC, MKN-45/BGN/NC and NCL-N87/NC groups. (D–F) Bar charts show numbers of tubules (D), intersecting nodes (E) and mean tubular lengths (F) between different groups. Data are represented as mean±SD of three independent experiments. *P< 0.05; **P < 0.01 compared with controls group.

We collected supernatants from SGC-7901/vector and SCG-7901/BGN to suspend human umbilical vein endothelial cells (HUVECs). Tubular formation ability of HUVECs treated with each cell types supernatant was assessed under microscope after 8 h incubation at 37°C with 5% CO_2_. The SCG-7901/BGN cells strong promoted tubular formation of HUVECs compared with control cells (SGC-7901/vector) (Fig.4A) in tubular number (Fig.4D), tubular intersecting nodes (Fig.4E) and tubular length (Fig.4F). We obtained opposite results after BGN was knocked down (Fig.4A, D, E, F). BGN expression in MKN-45 cancer cells was upregulated and then knocked down to examine the reproducibility of the finding. As expected, we got similar results (Fig.4B, C, D, E). NCL-N87 cells with knocked-down BGN showed similar results with SGC-7901/BGN/sh1 and MKN-45/BGN/sh1 cells (Fig.4C, D, E, F).

**Fig.5:**
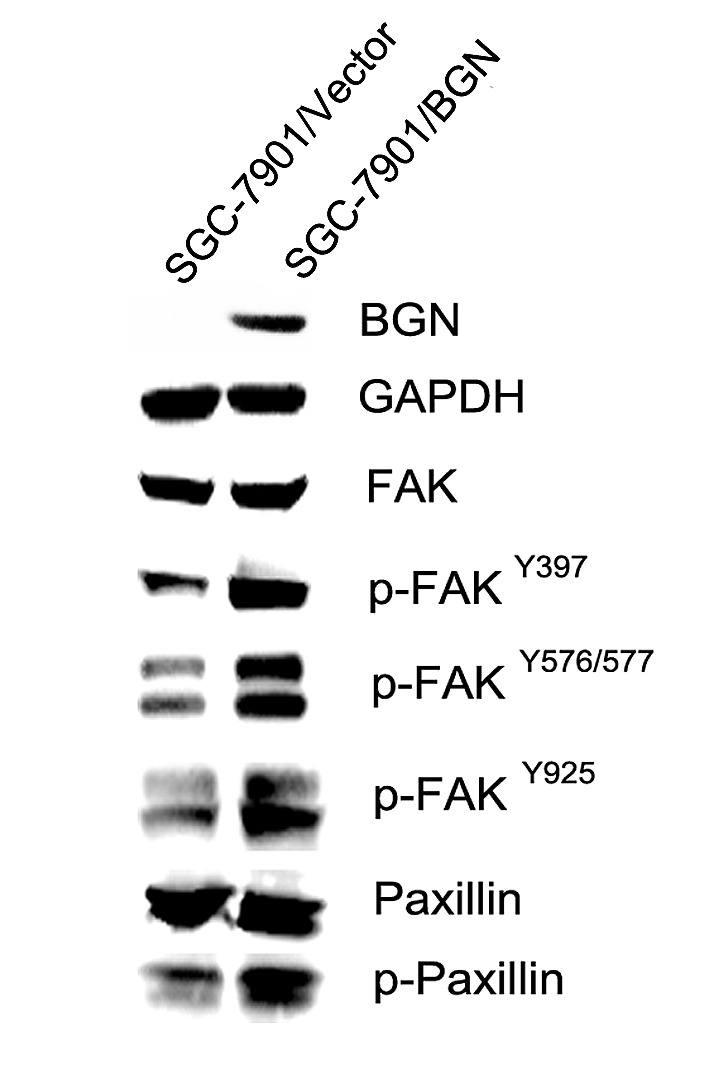
BGN regulates FAK signaling in gastric cancer cells Western blot analysis of FAK, phosphorylated FAK and its downstream protein paxillin or phosphorylated paxillin in the cytosolic fraction. GAPDH was used as a loading control.

### BGN promotes peritoneal spreading *in vivo*

**Fig.6:**
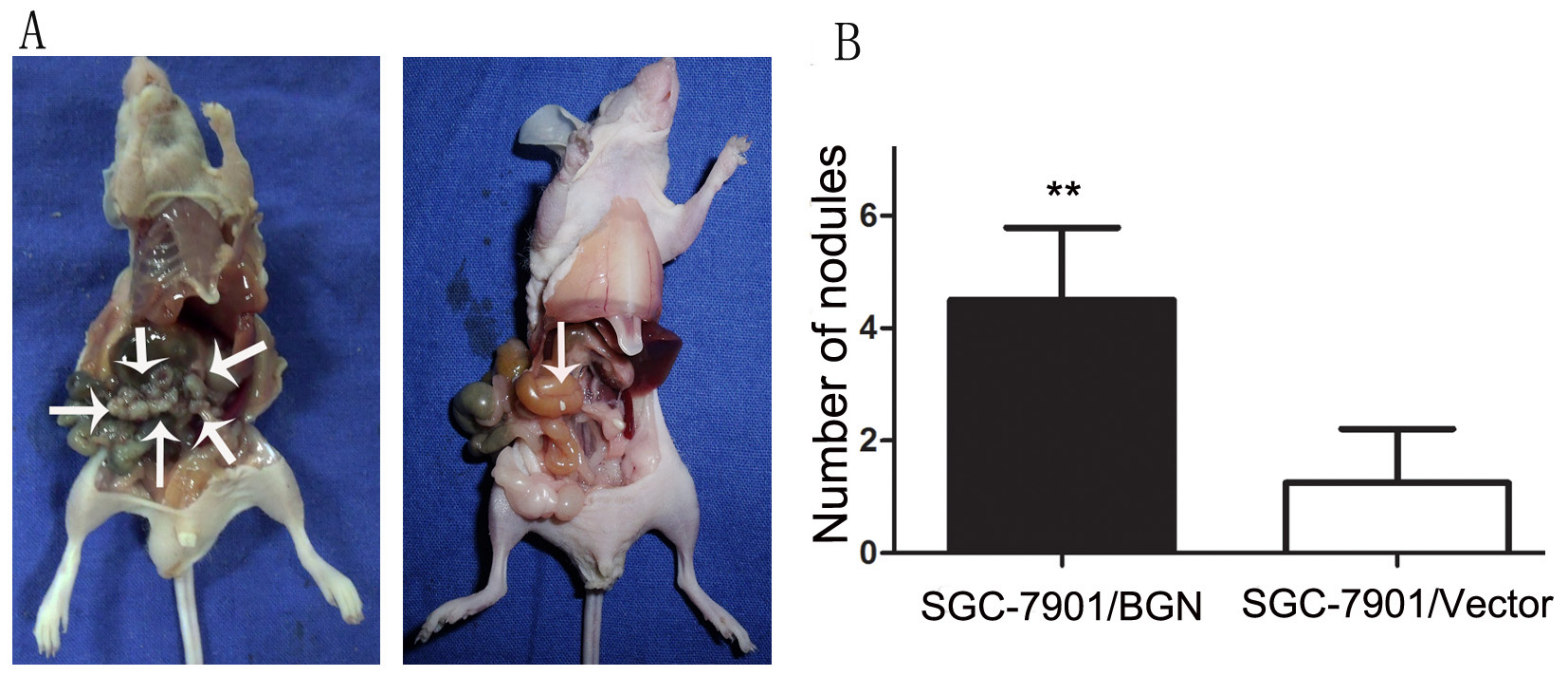
Effect of BGN on peritoneal spreading and metastasis (A) The peritoneal nodules were observed and photographed after the mice was sacrificed. (B) Bar charts show the average number of peritoneal nodules in each mouse.

Based on the *in vitro* findings described above, we examined the *in vivo* effect of over-expressed BGN by injecting SGC7901/BGN and SGC7901/Vector cells subcutaneously into abdomens of nude mice. As expect, there were significantly more visible peritoneal nodules in SGC-7901/BGN group than the control group (4.500±1.291 vs 1.250±0.957, *P*<0.05; Fig.6B). Extensive peritoneal spread was observed in the SGC-7901/BGN group compared with SGC-7901/Vector group (Fig.6A). These *in vivo* data were consistent with the *in vitro* results and confirmed that BGN overexpression promotes gastric cancer metastasis.

## DISCUSSION

Gastric cancer is a common disease worldwide, with a poor prognosis and low survival rates. Expression of the *BGN* gene was found to be higher in carcinoma tissues than in normal tissues [8, 9, 11, 16, 17]. Typically, high BGN expression implies poor prognosis. For example, BGN up-regulation was significantly correlated with poor tumor differentiation, lymph node metastasis, and distant metastasis in colorectal cancers, but not with other clinicopathological factors [18]. To our knowledge, this is the first report on the role of this gene in gastric cancer cells.

In the present study, we investigated the role of BGN in gastric cancer metastasis, *in vitro* and *in vivo*.Speed of wound healing was significantly increased in *BGN*-transfected gastric cells and significantly decreased after transfecting with *BGN* shRNA. In the transwell assay, more SGC-7901/BGN cells, MKN-45/BGN cells and NCL-N87 cells migrated to the lower chamber than control groups, which suggests that BGN enhances migration and invasion in gastric cancer cells. Xenograft model results *in vivo* supported the experimental outcomes *in vitro*. As with our findings, BGN knockdown inhibits tumor endothelial cell migration and tube formation in other studies [11]. BGN-enhanced migration is also observed in vascular smooth muscle cell [19]. Interestingly, we observed in PANC-I cells that BGN upregulation parallels changes in morphology and gene expression associated with TGF-β-induced epithelial–mesenchymal transition (EMT), such as acquisition of a spindle-shaped morphology and down- and upregulation, respectively, of epithelial and mesenchymal marker proteins [20, 21]. Hence, BGN may serve as another mesenchymal marker for EMT. What is even more interesting is that TGF-β-induced EMT requires focal FAK signaling [22]. Many studies have reported elevated expression of FAK protein in tumors, associated with increased rates of both migration and invasion [23, 24]. For example, inhibition of FAK apparently resulted in decreased cellular migration and invasion in neuroblastoma cell lines, and decreased metastasis in a murine model [25]. FAK regulates cell adhesion and motility by relaying ECM signals from integrins to the intracellular compartment [26]. FAK activation leads to the autophosphorylation of Tyr 397, creating a binding site for src. Subsequent binding of FAK to src lead to the formation of an active and transient FAK–src signaling complex, that in turn promotes src-mediated phosphorylation of FAK within the kinase domain activation loop (Tyr576 and Tyr577), and phosphorylation of FAK at the C-terminal domain residues, Tyr861 and Tyr925 [27]. Studies have shown a novel role for FAK activity in promoting a MAPK-associated angiogenic switch during tumor progression [28]. Paxillin is a substrate for the FAK–Src complex that functions as an adaptor molecule for various signaling and structural proteins in adhesions. Phosphorylated paxillin binding to the SH2/SH3 adaptor protein Crk is implicated in Rac activation and stimulation of cell motility [29]. Our study shows that BGN induces increased FAK phosphorylation (Tyr576/577, Tyr925 and Tyr397) and Paxillin phosphorylation (Fig. 5 and Fig. S2), which in turn may alter migration rates and invasion (Fig. 3).

In conclusion, we found that BGN expression in gastric cancer tissues was significantly upregulated compared with its adjacent normal gastric tissues, and was correlated with the clinical features of axillary lymph node metastasis, depth of tumor invasion and TNM stage. Moreover, BGN overexpression promoted migration and invasion of gastric cancer cells *in vitro* and in transplanted tumor models. It may serve as a useful indicator for tumor metastasis, and its effects might be partially mediated by modulation of the FAK pathway.

## MATERIALS AND METHODS

### Patients and tissue specimens

This study was approved by the Ethics Committee of Shanghai Ruijin Hospital, and all patients were fully informed of the experimental procedures. From 2009 to 2012, 122 sets of gastric tumor and adjacent non-tumorous tissues (at least 5 cm away from the tumor margin) were obtained from patients who underwent curative surgery at Shanghai Jiaotong University School of Medicine Affiliated Ruijin Hospital. They were 92 men and 30 women, with a mean age of 60.1 years (range: 30–78 years). None of the patients had received radiotherapy or chemotherapy before surgery. Clinicopathological data were collected and pathological tumor staging was determined according to the UICC TNM classification. Histological typing was performed by at least two expert pathologists, working independently in a double-blinded fashion.

### Cell lines

The human gastric cancer cell lines SGC-7901, MKN-45 and NCL-N87 were purchased from Shanghai Institutes for Biological Sciences, Chinese Academy of Sciences. PRMI 1640 containing 10% calf serum, 100 IU/ml penicillin and 100 IU/ml streptomycin were used as conventional culture medium. The culture procedures were taken under 37°C, 5% CO_2_ and saturation humidity.

### Total RNA isolation and quantitative realtime polymerase chain reaction

Total RNA was extracted and isolated from tissue samples or cell lines using Trizol reagent (Invitrogen), according to the manufacturer's instructions. Reverse transcription of RNA was carried out using the reverse transcription kit (Promega, Madision, WI, USA). In brief, 1 ug of total RNA from each sample was reverse transcribed following the manufacturer's recommended protocol. SYBR Green reagent (Applied Biosystems, CA, USA) was used for qRT-PCR to analysis mRNA expression. The PCR primers were designed for *BGN* were 5′- TTTGAGCAGAGAGGCTTCTGG-3′ (forward) and 5′- AAAGGACACATGGCGCTGTAG-3′ (reverse); for *GAPDH* were 5′-GGACCTGACCTGCCGTCTAG-3′ (forward) and 5′ -GTAGCCCAGGATGCCCTTGA-3′ (reverse) according to the human *BGN* and *GAPDH* cDNA sequences in GenBank. The PCR reactions were carried out at 95°C for two minutes, then a 3-step cycle procedure was used (denaturation at 95°C for 10 s, annealing at 60°C for 20 s, and elongation at 72°C for 40 s) for 40 cycles, with a final extension at 72°C for 10 min. *GAPDH* served as the constitutive control. PCRs of each sample were conducted in triplicate. Relative expression ratios of *BGN* in each paired tumor to non-tumor tissue sample were calculated using the 2^−ΔΔCt^ method. Up-regulation of *BGN* was considered to be positive in tumor tissue only when the BGN expression score was > 1.7 [13].

### Western blot analysis

Cells were digested in RIPA buffer in the presence of Protease Inhibitor Cocktail (Pierce, Rockford, USA). Protein was quantified using a BCA Protein Assay Kit (Pierce, Rockford, USA). Protein (100 ug) was separated by 10% sodium dodecyl sulfate polyacrylamide gel electrophoresis, and transferred onto polyvinylidene fluoride membranes. The membranes were blocked with 5% non-fat milk in Tris-buffered saline and then incubated with primary antibodies at 4°C overnight. The primary antibodies used were anti-BGN (1:1000; Abcam, USA), anti- focal adhesion kinase (FAK) (1:1000; Cell Signaling Technology, USA), anti-p-FAK (Tyr576/577, 1:1000; Cell Signaling Technology, USA), anti-p-FAK (Tyr925, 1:1000; Cell Signaling Technology, USA), anti-p-FAK (Tyr397, 1:1000; Cell Signaling Technology, USA), anti-paxillin (1:1000; Cell Signaling Technology, USA), anti-p-paxillin (1:1000; Cell Signaling Technology, USA) and anti-GAPDH (1:10000; Abcam, USA). Membranes were then washed three times in TBST solution for 15 min each time, and then incubated with secondary antibodies. Signals were detected by an enhanced chemiluminescence detection system (Amersham Bioscience, Piscataway, NJ, USA) as the manufacturer's protocol.

### Immunohistochemistry

Paraffin-embedded tissue sections from gastric cancer specimens were given a heat pretreatment of 60°C for one hour, then dewaxed in xylene, re-hydrated in an ethanol series (100–50%) and treated in 0.01 mol/L citrate buffer (pH 6.0) for antigen retrieval. After inhibition of endogenous peroxidase activity for 30 min with methanol containing 0.3% H_2_O_2_, the sections were stained with a mouse anti-BGN monoclonal antibody (Abcam, dilution 1:300) at 4°C overnight. The following experimental procedure was according to the manufacturer's instructions of the LSAB+ kit (Dako, USA). The cytoplasm was counterstained with hematoxylin. Two pathologists who were blinded from any patient data independently examined the cellular location of BGN and compared the staining between the tumor and normal tissues for each case. Immunohistochemistry stain score = positive cell score + staining intensity score [14]. The percentage of positive cells was classified by five grades (percentage scores): <10% (grade 0), 10–25% (grade 1), >25–50% (grade 2), >50–75% (grade 3), and >75% (grade 4). Immunohistochemical staining intensity was graded as follows: 0 (no staining), 1 (bright yellow), 2 (orange), or 3 (brown). The total scores of ≤2, >2–5, and ≥6 were defined as negative, weak positive, and strong positive, respectively.

### BGN overexpress cells construction

The full length cDNA of BGN was obtained by RT-PCR from total RNA of human gastric cancer tissues. The primers for the CDS double strand DNA fragments of *BGN* were 5′-CCGGAATTCGCCATGT GGCCCCTGTG-3′ (forward), 5′-CGCGGATCCCTGCAGCTG CCTCTACTTTTTG-3′ (reverse) and subcloned into pIRES2-EGFP vector to generate the pIRES2-EGFP/BGN construct. Transfection of the constructed plasmid and empty vector into SGC-7901 and MKN-45 cells was performed using Lipofectamine 2000 (Invitrogen) in accordance with the manufacturer's advised procedure. Cells that had been transfected with the constructed plasmid were then selected by antibiotic resistance in cell culture medium containing 1500 ug/ml G418 to obtain the cell strains with stable expression of BGN. After 6 weeks of culture in the presence of G418, the remaining cells were isolated and transferred into 24-well dishes. Positive clones were obtained in the pIRES2-EGFP/BGN vector transfection group (SGC-7901/BGN and MKN-45/BGN) and pIRES2-EGFP empty vector transfection group (SGC-7901/ Vector and MKN-45/Vector). The selected clones were taken for identification and frozen for future use.

### BGN knock-down cells construction

The shRNA of BGN were purchased from Shanghai GenePharma Co., Ltd. The shRNA duplexes and scrambled control duplexes were synthesized as follows:

The *BGN* shRNA duplexes:

S1 forward: CACCGGAGAACAGTGGCTTTGAA CCTTCAAGAGACGTTCAAAGCCA CTGTTCTCCTTTTTTG

S1 reverse: GATCCAAAAAAGGAGAACAGTG GCTTTGAACCTCTCTTGAAGGTTCA AAGCCACTGTTCTCC

S2 forward:

CACCGCCAGATCAGGATG ATCGAGAATTCAAGAGATTCTCGATCAT CCTGATCTGGTTTTTTG

S2 reverse: GATCCAAAAAACCAGATCAG GATGATCGAGAATCTCTTGAATTCT CGATCATCCTGATCTGGC Scrambled control duplexes :Forward: CACCGTTCTCCGAACGTGTCA CGTTTCAAGAGAACGTGACA CGTTCGGAGAATTTTTTG Reverse: GATCCAAAAAATTCTCCGA ACGTGTCACGTTCTCTTGAAACG TGACACGTTCGGAGAAC

A day before transfection, the SGC-7901/BGN, MKN-45/BGN and NCL-N87 cells were seeded onto six-well plates at 3×10^5^ cells per well. After cell culturing for 24 h when the cell density was ~70%, the cells were transfected with shRNA by Lipofectamine 2000, according to manufacturer's protocol. At 48 h post-transfection of the synthesized shRNA, the cells were used for identification and research.

### Immunofluorescence

Cells were seeded onto dedicated slides and cultured at 37°C for 24 h. Cells were then fixed with 4% paraformaldehyde. Cells on slides were next permeabilized with 0.1% Triton X-100 for 5 min at room temperature and then incubated overnight with primary antibodies (Abcam, dilution 1:200) at 4°C, followed by incubation with fluorescent secondary antibody for 1 h at room temperature. After final washes with PBS, the slides were mounted using an anti-fade mounting solution containing DAPI. Slides were analyzed and imaged on a fluorescence microscope.

### Wound healing assay

Cells were seeded in six-well plates and cultured until they reached confluence. Wounds were scratched on the monolayer of cells using 20 μL pipette tips. Plates were washed once with fresh medium to remove non-adherent cells after the cells had been cultured for 0, 24 or 48 h, and then photographed.

### Transwell migration and invasion assay

For the migration assay, 1×10^5^ cells were suspended in serum-free RPMI-1640 and plated on chambers (Corning Costar, NY, USA) that were not coated with Matrigel. For the invasion assay, the upper chamber was precoated with Matrigel (BD Bioscience, CA, USA) according to the manufacturer's protocols before 1×10^5^ cells in serum-free RPMI-1640 were added to the chamber. For both assays, medium containing 10% FBS was added to the lower chamber as a chemoattractant. After 24-h culture, chambers were stained with 0.5% crystal violet solution for 15 min, and then immersed in phosphate-buffered saline (PBS) for 10 min. Finally, cells in the lower chamber were counted under an inverted microscope. Values are expressed as mean cell numbers in 5 random fields of view (200×).

### Endothelial tube formation assay

Briefly, each well of prechilled 96-well culture plates was coated with a thin layer of the Matrigel (BD Biosciences, CA, USA), which was left to polymerize at 37°C for 1 h. human umbilical vein endothelial cells (HUVECs) were resuspended in collected supernatants from each cell type, and were added (2×10^4^ cells/well) to the polymerized Matrigel with 300 ul of the supernatants. After 8 h incubation at 37°C with 5% CO_2_, tube forming ability was evaluated by counting tubes, their length and the number of tubular intersecting nodes in 5 random fields using Image Pro Plus software according to Mirshahi's method [15].

### Xenograft model

Four-week-old male BALB/C nude mice were purchased from the Institute of Zoology, Chinese Academy of Sciences of Shanghai. All experiments were performed in accordance with the official recommendations of the Chinese animal community. Gastric cancer xenografts were established in nude mice. Briefly, SGC-7901 cells that stably expressed empty vector or BGN were trypsinized and resuspended in PBS (pH 7.4) for injection into one mouse in a total volume of 100 ul. The suspension, containing 1×10^6^ cells, was injected into the abdominal cavity (five in each group). On the 30th day after intraperitoneal injection, mice were sacrificed by cervical decapitation. Peritoneal spreading and metastasis were then observed and photographed.

### Statistical Analysis

Data are shown as mean±SD. Statistical differences between the two groups were examined by Student's *t*-test. Correlations between BGN expression in gastric cancer tissues and clinicopathological parameters were analyzed by chi-square or Fisher's exact tests. *P*<0.05 was considered significant, and *P*<0.01 was considered highly significant.

## SUPPLEMENTARY FIGURE


