# Supplementary figures and images for: Biglycan enhances gastric cancer invasion by activating FAK signaling pathway
Source: Oncotarget. 2014 Mar 26;5(7):1885–96. doi: 10.18632/oncotarget.1871 (PMC4039113; doi:10.18632/oncotarget.1871)

Biglycan enhances gastric cancer invasion by activating FAK signaling pathway

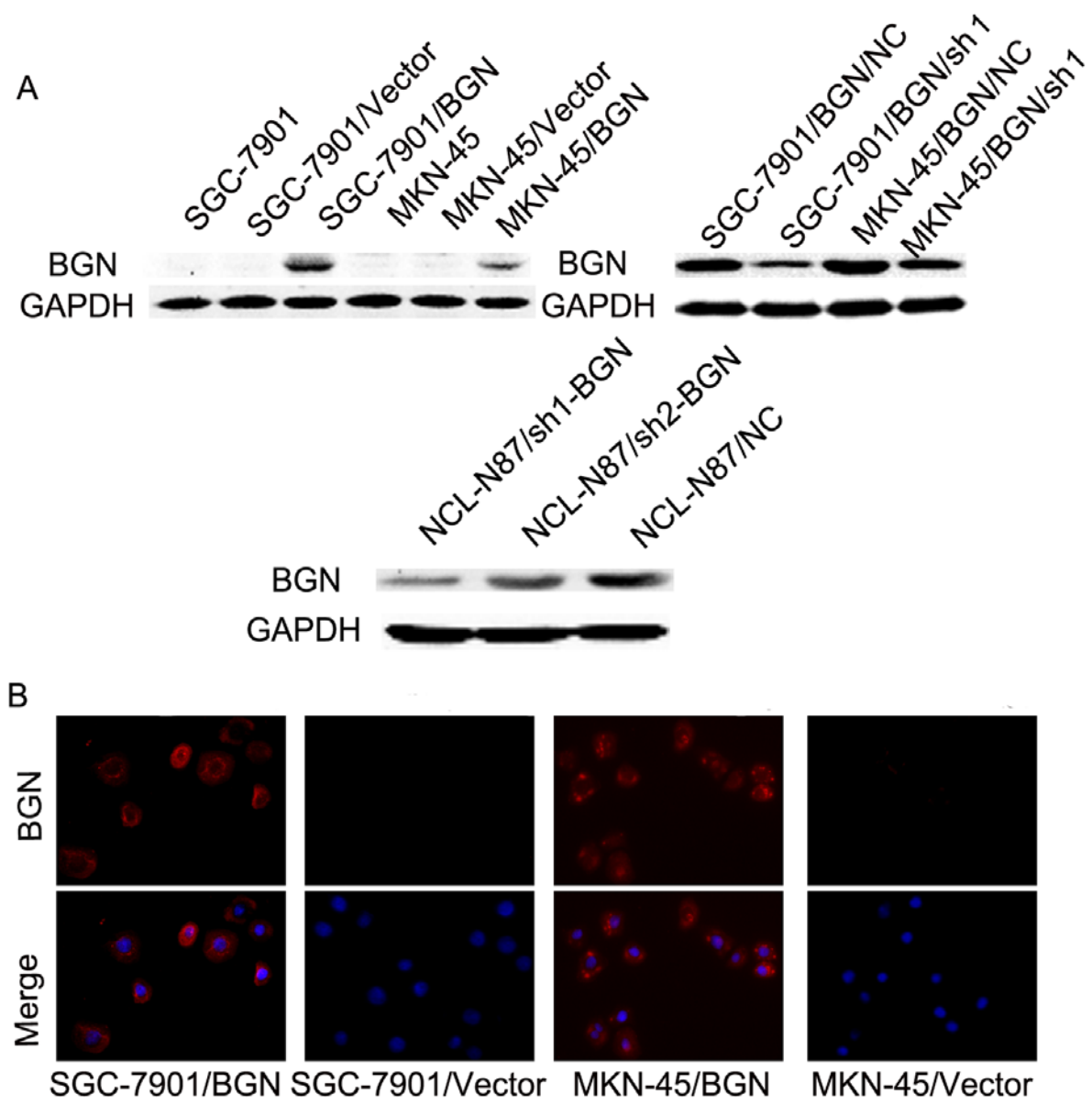

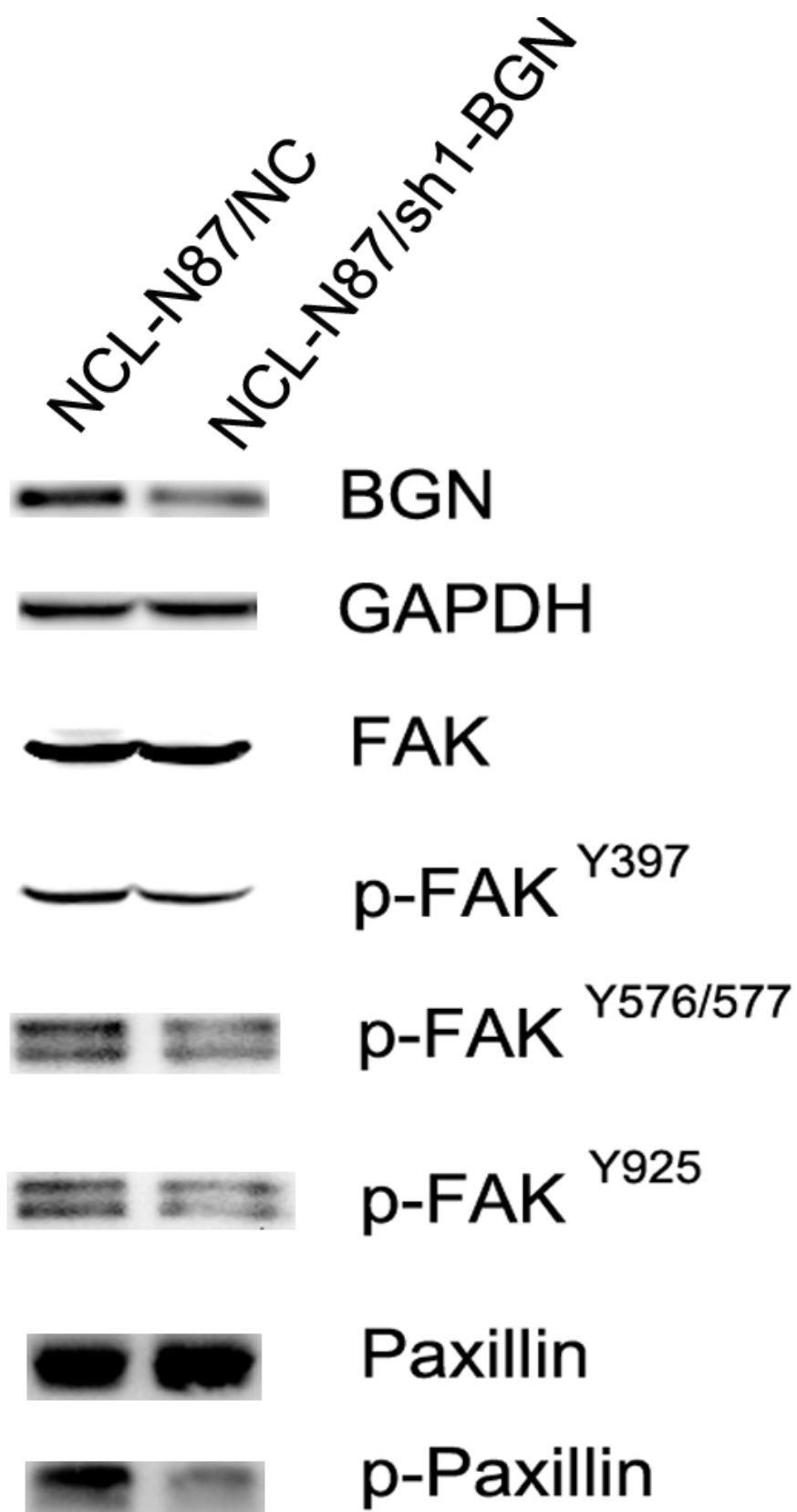

Supplement: Supplementary file 1 [file oncotarget-05-1885-s001.pdf]
